# Interaction of natural and hydraulic fractures: the impact on reservoir pressure buildup and risk of shear fault reactivation in the Upper Devonian Duvernay Formation, Fox Creek, Alberta

**DOI:** 10.1007/s40948-023-00537-z

**Published:** 2023-02-27

**Authors:** Qiuguo Li, Elena Konstantinovskaya, Alexey Zhmodik, Charles Ibelegbu

**Affiliations:** 1 Saudi Aramco, Dhahran, Saudi Arabia; 2 Earth and Atmospheric Sciences, University of Alberta, 1-26 Earth Sciences Building, Edmonton, AB T6G 2E3 Canada; 3 Subsurface Dynamics Inc., Calgary, AB Canada; 4 Resourcesfusion Ltd, Calgary, AB Canada

**Keywords:** Discrete fracture network, Hydraulic fracturing, Induced seismicity, Shear slip fault reactivation, Reservoir-geomechanical modeling, Duvernay Formation, Western Canada Sedimentary Basin

## Abstract

**Abstract:**

The interaction of natural and hydraulic fractures may facilitate lateral fluid propagation in an unconventional reservoir resulting in fast fluid pressure transmission from treatment wells to a fault zone and potential fault shear slip reactivation and associated induced seismicity. Several induced earthquakes (up to 4.1 Mw) occurred since 2013 during hydraulic fracturing of the Upper Devonian Duvernay Formation in the Western Canada Sedimentary Basin. The mechanism of lateral fluid migration in the unconventional reservoir is not well understood. The current study aims to investigate the interaction of natural fractures and hydraulic fractures for the case study in the area south of Fox Creek, where a linear zone of induced earthquakes (up to 3.9 Mw) occurred along a fault in 2015 during hydraulic fracturing of horizontal wells. We analyze the growth of hydraulic fractures in presence of natural fractures, the impact of resulting complex fracture network on fluid transmission and fluid pressure buildup around the treatment wells. Hydraulic fracture modeling (HFM), reservoir simulations and 3D coupled reservoir-geomechanical modeling are applied to match the timing of hydraulic fracture propagation and transmitted fluid pressure increase in the fault zone versus induced earthquake occurrence. HFM results are verified by microseismic clouds distribution. Reservoir simulations are validated by a history matching of fluid injection volume and bottomhole pressure data. Additional HFM simulations are carried out to optimize the pumping schedule in the studied well pad that would help to prevent hydraulic fractures reaching the fault and minimize the risk of induced seismicity.

**Article highlights:**

- Stress anisotropy and simulated natural fractures impact lateral growth of complex hydraulic fractures and reservoir pressure buildup.
- Predicted fluid pressure transmission to a fault zone results in fault dextral shear slip reactivation matching induced seismicity.
- Optimized pumping schedule helps to minimize risks of fault reactivation and induced seismicity while preserving overall pad performance.

## Introduction

**Fig. 1:**
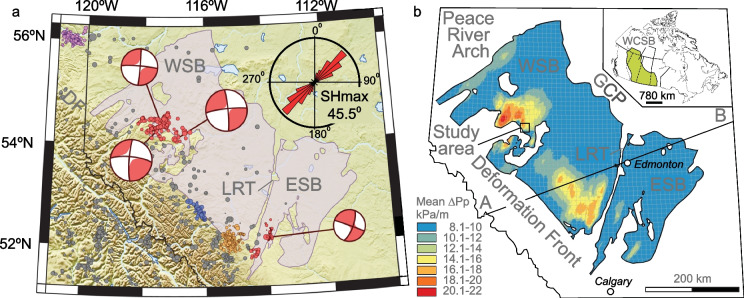
Map of the Duvernay Formation in the West Shale Basin (WSB) and East Shale Basin (ESB) of the Western Canada Sedimentary Basin (WCSB), displaying: **a** locations of HF‐induced earthquakes are shown by red circles, after (Schultz et al. 2020); focal mechanisms of the four largest events are shown, from left to right: 12 January 2016, 4.1 Mw; 23 January 2015, 3.6 Mw; 13 June 2015, 3.9 Mw; and 4 March 2019; orientation of present-day maximum horizontal stress (SHmax) is shown after (Reiter et al. 2014); **b** mean simulated fluid pressure gradient (mean ΔPp, kPa/m) in the Duvernay Formation, after (Lyster et al. 2017). *GSP* Grosmont carbonate platform; *LRT* Leduc Rimbey Meadowbrook Reef Trend; *DF* Deformation front. See Fig. 3 for cross-section A–B

Lateral fluid migration in subsurface reservoirs during hydraulic fracturing is affected by several parameters, including reservoir pressure, stress anisotropy, bedding and mechanical properties of rocks, and interaction of natural (NFs) and hydraulic fractures (HFs). Numerical simulations studies have shown that presence of natural fractures in a formation impacts hydraulic fracture propagation resulting in complex HFs–NFs networks (Weng et al. 2011; Wu et al. 2012; Haege et al. 2015; Weng 2015; Rutqvist et al. 2018). The fracture network complexity contributes to the efficiency of hydraulic fracturing operations in unconventional reservoirs (Ramanathan et al. 2015; Ferrer et al. 2020; Li et al. 2020). Additionally, fluid pressure transmission through complex HFs–NFs networks in tight fractured formations may result in shear slip reactivation of critically-stressed faults and induced seismicity. It has been shown that induced seismicity in the Western Canada Sedimentary Basin (Fig. 1a) in several cases occurred during and/or shortly after hydraulic fracturing of the Upper Devonian Duvernay Formation (Eaton et al. 2018; Schultz and Wang 2020; Schultz et al. 2020). However, the processes that result in induced seismicity and mechanisms of fault reactivation are still poorly understood and require further development (Eyre et al. 2019; Igonin et al. 2021; Konstantinovskaya et al. 2021). In this study, we analyze the impact of NFs represented by the Discrete Fracture Network (DFN), on simulated complex fracture networks and lateral fluid pressure transmission and the potential of fault shear slip reactivation in the Fox Creek area, Alberta.

The Upper Devonian Duvernay Formation is an organic-rich shale formation (Fig. 1a) in the Western Canada Sedimentary Basin (WCSB). It is source rock for historic conventional production in Leduc and Swan Hills carbonate reef reservoirs, and it is being developed as an unconventional shale resource (Preston et al. 2016). The development of Duvernay started in 2011 with horizontal wells and multistage hydraulic fracturing (HF). The horizontal wells are commonly completed with limited entry plug and perf designs.

Induced seismicity in the WCSB has previously been attributed to stress changes from hydrocarbon production, enhanced oil recovery, and wastewater disposal. Most recent occurrences of earthquakes are believed to be associated with hydraulic fracturing in the unconventional reservoirs (Atkinson et al. 2016; Schultz and Wang 2020). The largest events occurred in 2015–2019 (Fig. 1a) are characterized by magnitude 3.7–4.1 Mw and sub-longitudinal dextral strike-slip earthquake mechanism (Schultz et al. 2020). In the Fox Creek area, large induced earthquakes are mostly located above the Duvernay Formation, in the Ireton carbonate-rich mudstones and Wabamun carbonate rocks (Eaton et al. 2018; Konstantinovskaya et al. 2021).

**Fig. 2:**
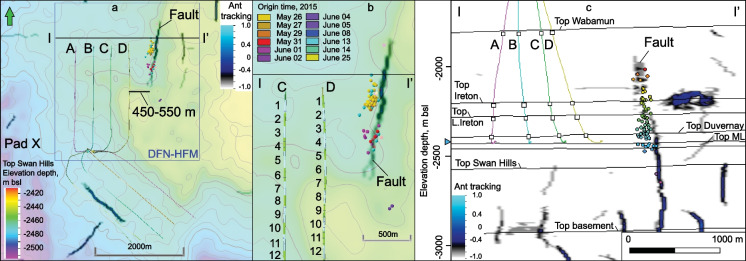
**a** Map of ant tracking attribute at depth 2425 m bsl in the Duvernay Formation and structural depth map of the top of the underlying Swan Hills Formation in the study area south of the Fox Creek, West Shale Basin. Linear discontinuities in ant-tracking attribute volume are highlighted by dark blue color and may correspond to faults. Polygon shows the area of DFN-HF modeling. **b** Location of linear N-S zone of induced earthquakes east of the treatment wells A–D (Pad X). The earthquakes (up to 3.9 Mw) are colored according to their origin time. **c** Cross-section of ant-tracking attribute volume along the W–E line I–Iʹ. Wells A–D are projected orthogonally on the cross-section I–Iʹ over the distance of ~ 0.2–2 km. Blue arrow indicates depth slice at 2425 m bsl (**a, b**). The earthquakes are colored according to the depth of hypocenters. L. Ireton, Lower Ireton; ML, Majeau Lake

We investigate a case study of induced earthquakes that occurred in May–June 2015 in the Kaybob area of the West Shale Basin, approximately 30 km south of Fox Creek (Fig. 1b). The earthquakes were recorded from May 26 to June 25 of 2015, during hydraulic fracturing of the Duvernay Formation in a well pad X in the area (Fig. 2). The earthquakes occurred along a N–S linear zone, approximately 450 m to 550 m east of the nearest horizontal well on the well pad (Schultz et al. 2017; Konstantinovskaya et al. 2021). The induced events occurred first in a northern segment of the zone, followed by events in its southern segment, and finally in both segments (Fig. 2b). The highest magnitude of the earthquakes of 3.9 Mw occurred on June 13, 2015.

In this paper, we study the impact of geometry of natural fractures (DFN) in the Duvernay Formation on the growth of hydraulic fractures and lateral fluid migration around the treatment wells to match the timing of HFs propagation and increase of fluid pressure transmitted to the fault zone versus occurrence of induced earthquakes. Additionally, a sensitivity study on HFM simulations is carried out to optimize the pumping schedule in the well pad that would prevent hydraulic fractures reaching the fault and help to minimize the risk of induced seismicity.

## Background information

**Fig. 3:**
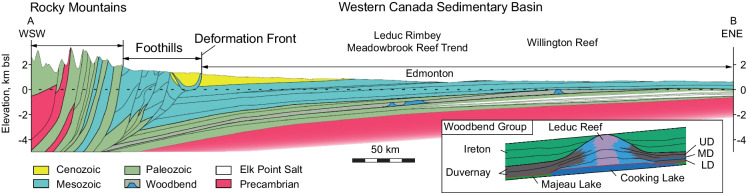
Geological cross-section A–B of the Western Canada Sedimentary Basin, modified after (Hamilton et al. 1999; Reiter and Heidbach 2014). Vertical exaggeration 10x. See Fig. 1b for the cross-section location. Inset section displays composition of the Woodbend Group and the Duvernay depositional model, modified after (Dunn et al. 2013). UD, MD, LD, upper, middle and lower Duvernay, respectively

The Duvernay Formation is recognized in the West and East Shale Basins that are separated by the Leduc Rimbey Meadowbrook Reef Trend (Figs. 1a, 3). The study area is located in the West Shale Basin (Fig. 1b). The Duvernay Formation consists of upper and lower organic-rich shale units separated by carbonate-rich lean shale middle unit, and it is overlain by the Ireton Formation and underlain by the Majeau Lake and Swan Hills Formations (Fig. 3). The top of the Duvernay Formation gradually dips from about 1 km in the NE to more than 5 km in the SW. In the study area, it is approximately 3350 m deep, and the total thickness increases eastward from 25 to 72 m, averaging ~ 50 m. Porosity ranges from 3 to 8%, and permeability varies within from 70 to 150 nD (Kleiner and Aniekwe 2019; Konstantinovskaya et al. 2021).

The mean orientation of the present-day maximum horizontal stress SHmax in the WCSB (Fig. 1a) is N45.5° E as estimated from the borehole breakouts (Reiter et al. 2014) and it is N43° E in the Fox Creek area (Shen et al. 2019a, b). The conjugate planes of the induced earthquake focal mechanisms strike predominantly N–S and E–W (Fig. 1a) and indicate strike-slip displacement with P-axis vectors at an azimuth of N45° E ± 5° (Schultz et al. 2017, 2020) that is consistent with regional horizontal stress orientation.

The present-day stress regime in the WCSB varies from thrust faulting in the Foothills of the Rocky Mountains (Fig. 3) deformed belt to strike slip within the basin to normal faulting further east in Saskatchewan (Reiter and Heidbach 2014). The Kaybob area of the WCSB is generally characterized by the strike-slip fault regime with maximum horizontal stress (SHmax) greater than vertical stress (Sv), which is greater than minimum horizontal stress (Shmin) (Shen et al. 2019a, b). In the study area south of Fox Creek (Konstantinovskaya et al. 2021), the Sv gradient in the Duvernay Formation is approximately 25–25.5 kPa/m. Magnitude of horizontal stresses estimated by applying the poroelastic horizontal strain model (Thiercelin and Plumb 1994) vary depending on mechanical stratigraphy of the sedimentary rocks. SHmax tends to concentrate on stronger carbonate rocks above (Wabamun Group) and below (Swan Hills Formation) the Duvernay Formation, and it decreases in more ductile organic rich shales of the Duvernay Formation. The SHmax and Shmin gradients are 25.2–28.8 kPa/m and 22.8–25.2 kPa/m in the carbonate rocks, and they decrease to 21.5–22 kPa/m and 19.5–20.5 kPa/m in the Duvernay Formation, respectively. Fluid pressure gradient in the Duvernay Formation varies across the basin (Fig. 1b) and can be as high as 20 kPa/m (Lyster et al. 2017; Eaton and Schultz 2018), and it ranges between 16 and 17 kPa/m in the study area (Konstantinovskaya et al. 2021).

Both open and healed natural fractures (NFs) are recognized in the Duvernay Formation of the Kaybob area, and they are mostly high angle fractures (Fothergill et al. 2014). Three statistically significant sets of natural fractures were determined from image logs in two horizontal wells in the Kaybob area (Fothergill et al. 2014). Two of the three sets (strike azimuth N55° E and N130° E) were reported to be sub-parallel to maximum and minimum horizontal stresses, respectively. The third set (strike azimuth N75° E) was found to be oriented at 20° to the maximum horizontal stress in that area. Natural fractures previously studied in core and image logs of the Duvernay Formation are either open or calcite filled, dip steeply (75°–85°), and assumed being created during tectonic events (Kleiner and Aniekwe 2019). The measured fracture density is up to 8 fractures per meter, and average open fracture density is ~ 1–2 per meter.

In the study area, eight horizontal wells were drilled in the Duvernay Formation on well pad X (Fig. 2) with a well spacing of approximately 375 m. The four wells drilled toward north are the closest to the earthquakes. Well A and B were fractured in zipper mode from April 25 to May 11, 2015 with an average pumping rate of 13.2–13.5 m^3^ per minute. In wells C and D, 15 stages were fractured also in zipper mode from May 22 to June 5, 2015 with an average pumping rate of 14.3–14.6 m^3^ per minute. The four horizontal wells drilled toward the southeast were fractured at the same time. During fracturing of these eight wells, the maximum bottomhole pressure reached over 80 MPa. The induced earthquakes occurred to the east of well D (Fig. 2a) during and after the fracturing of Wells C and D, starting from May 26 (Fig. 2b, c).

Our previous seismic interpretation of 3D amplitude cube by applying ant tracking attribute analysis in the study area helped to identify a linear discontinuity zone located to the east of well D (Fig. 2) that may represent a fault (Konstantinovskaya et al. 2021). The ant tracking algorithm was developed by Schlumberger Stavanger Research and it is available in the PETREL™ software (Silva et al. 2005); the attribute is used to identify fault zones in volume and enhance horizon discontinuities. The interpreted fault extends for about 1.4 km along the strike and consists of northern and southern segments that are oriented at 33° to SHmax, and they are connected by a relay zone oriented at ~ 40–43° to SHmax (Fig. 2). In a cross-section, the fault can be traced for about 600 m from the top of Precambrian basement to Upper Devonian carbonate mudstones of the Ireton Formation, locally up to carbonate rocks of the Wabamun Group (Fig. 2). The fault is subvertical, with no visible offset of seismic reflections, and, given that seismic survey was acquired before hydraulic fracturing in the study area, it is interpreted as a pre-existing strike-slip fault. The epicenters of induced earthquakes are projected along the two segments with less events in the relay zone (Fig. 2).

### Data, methods and workflow

**Fig. 4:**
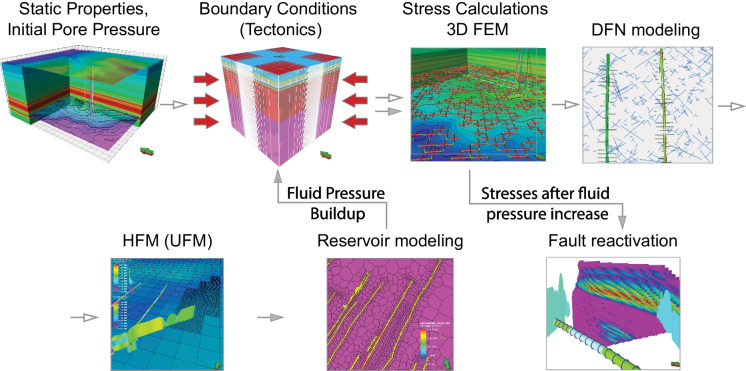
The workflow of 3D reservoir-geomechanical modeling used in this study. The white arrows indicate the pre-injection steps of the modeling under initial pressure and stress settings; filled arrows indicate the post-injection steps of the modeling after fluid pressure buildup in the reservoir around the treatment wells. DFN, Discrete Fracture Network; FEM, Finite Element Modeling; HFM, Hydraulic Fracture Modeling; UFM, Unconventional Fracture Modeling

The present study is based on our previous research that involved multidisciplinary approach (Konstantinovskaya et al. 2021). The pre-injection steps of the workflow (Fig. 4) include 3D structural modeling, 3D static geomechanical modeling, and DFN modeling carried under initial pressure and stress conditions. The subsequent steps of hydraulic fracturing and reservoir simulation of fluid pumping are performed to generate the system of complex HFs–NFs and simulate the reservoir pressure increase and its lateral and vertical propagation around the treatment wells. The 3D one-way coupled reservoir-geomechanical simulation is conducted for the post-injection steps aiming to estimate the stresses after the reservoir pressure increase during hydraulic fracturing and to analyze parameters that control fault shear slip reactivation.

The 3D structural model is built in Petrel™ (https://www.software.slb.com/products/petrel). The model dimension is 7.65 km × 8.55 km and elevation ranges from 1100 m asl to 3200 bsl. The total number of grid cells is 0.965 × 10^6^. The lateral cell size 150 m. Local grid for the area of fault zone has cell size 15 m × 15 m. The model contains 16 horizons including 14 interpreted horizons complemented by topography surface and a flat bottom horizon at depth 3200 m bsl. Horizons are layered into 333 layers in total. The number of layers in each zone is selected to generate upscaled logs close enough to recorded well logs. Cell height in targeted interval of the Duvernay Formation is 1.0–1.8 m that gradually increases to 10 m in the overlying Wabamun Group and to 10 m in the basement. The 3D geomechanical model is built by adding to the structural model sideburden and underburden to form a cube of 31.2 km size to eliminate side effects of model loading. The total grid cells number of the 3D geomechanical model is 1.82 × 10^6^. The lateral cell size gradually increases in the sideburden and underburden. The fault elements in 3D geomechanical grid are assigned as cells that are crossed by the fault plane interpreted from ant-tracking attribute (Fig. 2). The mechanical properties of rocks, initial pore pressure and three principal stresses are estimated in 1D and 3D geomechanical models and calibrated by core testing data and DFIT tests (Konstantinovskaya et al. 2021).

DFN modeling is conducted to simulate the presence of natural fractures in the Duvernay Formation. The DFN model was generated in Fracture modeling function in Petrel™. The parameters for two DFN models are described in the Results section. Orientations of natural fracture sets are modeled based on the previous analysis of natural fractures in borehole image logs in two horizontal wells of the Kaybob area (Fothergill et al. 2014). Additionally, we apply fracture trends obtained from the ant-tracking attribute for the depth interval of the Duvernay. NFs are simulated as vertical based on previous observations of high-angle fractures in core and image logs of the Duvernay Formation (Kleiner and Aniekwe 2019). Fracture intensity, length, aperture and permeability are determined by stochastic fracture model as no image log data in the study area is available. Fracture permeability is related to fracture aperture by the cubic law: Permeability (mD) = 1/12 × aperture^2^ (m) × conversion factor (1 mD = 10^–15^ m^2^). When the simulation files are generated, the units of all the parameters are converted to the units required by the simulators. After the simulations are completed, the results are imported back to the software and the units of all parameters are converted to the ones, which are associated with their templates. DFN models were converted to 2D fracture models in the horizontal plane for hydraulic fracture modeling.

Hydraulic Fracture Modeling (HFM) is carried out in KINETIX (https://www.software.slb.com/products/kinetix) to create complex fracture networks and subsequently an unstructured grid for fluid flow simulation. Hydraulic fractures are tensile fractures. Petrel takes into account the fracture effect by modifying both the connection factors for all connections intercepted by the fracture and X, Y and Z transmissibility multipliers for regions surrounding the one that hosts the fracture. The inter-cell and well-cell transmissibility multipliers are generated in Petrel in explicit models of fractures as functions of a set of correlations of combinations of grid block sizes, fracture length, the angle between the fracture and the grid block, the ratio of the fracture permeability and the grid block permeability, and the well position in the grid block. The results of HFM is compared for two cases, in which we analyze the impact of two different DFN models on HFs propagation and reservoir pressure buildup. Case 1 involves HF simulation in horizontal wells C and D previously carried out with DFN-1 model (Konstantinovskaya et al. 2021). Case 2 consists of a new reservoir simulation of four horizontal wells A, B, C and D completed with DFN-2 model. Case 3 is a sensitivity study, which has similar settings to Case 2 but the pumping schedule is optimized to prevent HFs reaching the fault zone located at about 450–550 m to the east from horizontal well D. Fluid volume and proppant mass in Case 3 are reduced in hydraulic fracturing stages 6 and 7, from which HFs propagated far to the east and reached the fault zone in Case 2. More details are discussed in the Results section.

The mechanical properties of rocks (Young’s modulus, Poisson ratio, Biot coefficient, tensile and unconfined compressional strength, angle of internal friction); reservoir pressure, total stresses Sv, SHmax, Shmin, and azimuth of SHmax, that were estimated in the 3D geomechanical model in the previous step, are imported in the HFM. The reservoir porosity, horizontal and vertical permeability, net to gross ratio, fluid saturation (oil, gas, water), multimineral composition (dolomite, limestone, quartz, clay components) and mechanical properties of rocks in the Duvernay Formation are modeled in the HFM volume based on core testing data in vertical wells located within 4 km from the well pad (Becerra Delmoral and Konstantinovskaya 2020; Konstantinovskaya et al. 2021). The core-based measured total porosity varies from 2.8 to 6.32% with median value of ∼ 4.8%, standard deviation 1.8%, median effective porosity 4.5%, standard deviation 1.6%; matrix minimum permeability of ∼86 nD, that is similar to the published ranges of these parameters (3–8%, averaging 5% and 70–150 nD) for this formation (Kleiner and Aniekwe 2019). The completion in wells A–D are available from the geoScout database (https://www.geologic.com/geoscout/). The total fluid volume, water injection rate and bottomhole pressure per stage are available from the AccuMap database (https://ihsmarkit.com/products/oil-gas-tools-accumap.html). The types of treatment fluids (Hybrid 30–50–150) and proppants (Badger Sand 30/50) were applied based on published data on the fluid system and proppants generally used in the Duvernay Formation in the study area (Leshchyshyn et al. 2016). Temperature gradient was set at 2.995 °C/100 m that is supported by bottomhole temperature data in the analyzed wells and by published regional data (Lyster et al. 2017).

Unconventional Fracture Model (UFM) is used to simulate complex hydraulic fractures, taking in account interaction of hydraulically induced fractures with natural fractures in the formation represented by the DFN-1 and DFN-2 models. The UFM simulates propagation of hydraulic fractures using a pseudo 3D (P3D) fracture model. The simulation of hydraulic fracture propagation is based on the theory of linear elastic fracture mechanics. The criterion for fracture propagation is by comparing the stress intensity factor at the fracture tip with the fracture toughness; the HF will propagate when the stress intensity factor at the fracture tip is greater than the fracture toughness. When a growing hydraulic fracture is intersected by a natural fracture represented by DFN, the interaction of hydraulic fracture and natural fracture is simulated based on a fracture crossing criterion, which determines whether the hydraulic fracture will stop, cross the natural fracture, or dilate and propagate along the natural fractures (Gu and Weng 2010; Weng et al. 2011; Gu et al. 2012; Wu et al. 2012). When a hydraulic fracture is approaching a natural fracture, the stresses on the opposite side of the natural fracture plane is calculated based on the far-field stresses and hydraulic fracture tip stresses. Factors affecting this stress calculation include the angle between the hydraulic fracture and the natural fracture, the friction coefficient and the direction and magnitude of the far-field stresses. The calculated stresses are then compared to the tensile failure condition and shear slip condition to see if the hydraulic fracture will propagate through the natural fracture or induce a slip in the natural fracture plane. Additionally, the UFM model also considers the "stress shadow" effect on each fracture exerted by the adjacent earlier fractures. Petrel takes into account the fracture effect by modifying both the connection factors for all connections intercepted by the fracture and X, Y and Z transmissibility multipliers for regions surrounding the one that hosts the fracture. The inter-cell and well-cell transmissibility multipliers are generated in Petrel in explicit fracture models as functions of a set of correlations combining grid block sizes, fracture length, the angle between the fracture and the grid block, the ratio of the fracture permeability and the grid block permeability, and the well position in the grid block.

For QC, simulated HFs geometry is compared to microseismic clouds related to the treatment of wells A–D as discussed below in Results. If the footprint of complex fracture network that is generated by the UFM is in agreement with the microseismic clouds in general, it verifies that the UFM is representative, and the generated the fracture network is realistic. Since the microseismic events are associated with slips of micro-fractures, and size and connectivity of the micro-fractures may vary from location to location, it is not possible to have an exact match. Additionally, uncertainty of the locations of smaller-magnitude microseismic events is generally higher than it is for the locations of larger-magnitude events. A consistent orientation alignment, lateral and vertical extent of simulated HFs with the microseismic events is considered a good match.

Reservoir simulation is conducted in INTERSECT covering both injection and production stages (https://www.software.slb.com/products/intersect) in the unstructured production grid that is generated from HFM. Well completions, pumping schedules and other input data were used in the hydraulic fracture simulations for four horizontal wells A–D. The default parameters of drainage relative permeabilities, black oil fluid model (PVT), and rock compaction are applied. The field management strategy included the rate of injection and bottomhole pressure for the injector stages in horizontal wells. Unstructured reservoir simulation is conducted by coupling of HFM with reservoir fluid and rock properties with three steps: (i) construction of production grid (unstructured); (ii) defining reservoir fluids and rock properties; (iii) simulating the fluid injection (Konstantinovskaya et al. 2021). Reservoir simulations are validated by a history matching of fluid injection volume and bottomhole pressure data. Several reservoir simulations models (Appendix 1) were built to account for observed data and reduce uncertainty in reservoir porosity and permeability parameters, fracture and matrix relative permeability.

The horizontal wells A and B were fractured from April 25, 2015 to May 11, 2015. The other two horizontal wells C and D were fractured from May 22, 2015 to June 5, 2015. Both pairs of wells were fractured using zipper-frac sequence, alternating fracturing stages from one to the other well for improved efficiency. In simulating the fracturing process, we used simulation time rather than real time (over 20 days), and stress shadows generated from earlier stages were applied to the surrounding later stages of fractures within the distance of 420 m.

**Fig. 5:**
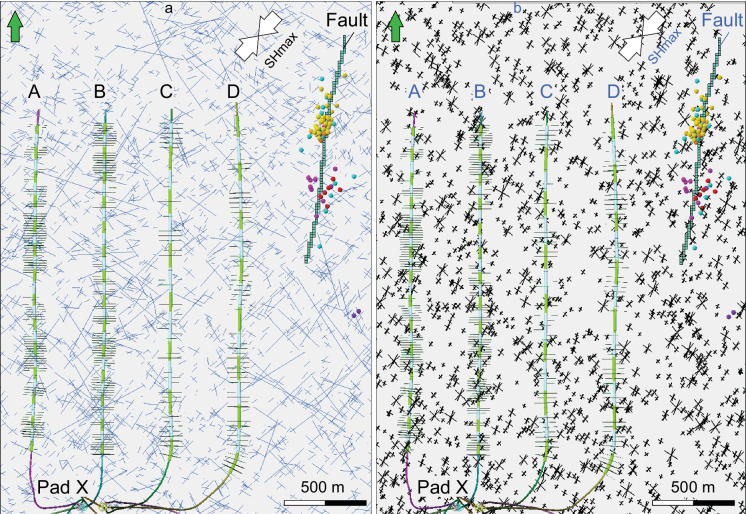
The 2D map view of DFN-1 (**a**) and DFN-2 (**b**) models with two and three sets of natural fractures, respectively. Displayed slice is parallel to a horizon located at the bottom of the Duvernay Formation. Fault elements (green color) correspond to fault location in 3D geomechanical grid. The induced earthquakes (up to 3.9 Mw) are colored according to their origin time (Fig. 2b)

3D one-way coupled reservoir-geomechanical finite-element modelling (FEM) involves iterative simulations and exchange of results between the geomechanical simulator VISAGE™ (https://www.software.slb.com/products/visage), and fluid flow simulator ECLIPSE (https://www.software.slb.com/products/eclipse) (Koutsabeloulis and Hope 1998). It allows to quantify magnitude of principal total stresses and orientation of horizontal stresses in each model cell. The post-injection stress estimation is conducted in VISAGE™, taking into account pressure changes, but HFs generated in KINETIX in previous steps are ignored. 3D coupled reservoir geomechanical modeling is performed to analyze fault mechanical instability as a result of fluid pressure increase transmitted from treatment wells to the fault zone through complex HFs–NFs. The fault in 3D geomechanical model was modeled as fault elements (Fig. 5), which are grid elements that are intersected by the fault surface interpreted from seismic volume to the east of the well pad (Fig. 2). The following fault properties are assigned to all fault elements: normal stiffness, 6 GPa/m, shear stiffness, 3 GPa/m, friction angle, 22.5 degrees, cohesion, 0.1 MPa, tensile strength, 0.01 MPa, and dilation angle, 0 degree. These values are estimated using the following equations: fault normal stiffness (Kn) [GPa/m] = YM-STA/t, where YM-STA is static Young Modulus of matrix material and t is the cell height [m]; fault shear stiffness (Ks) [GPa/m] = Kn/2; fault friction angle [deg] = 0.75 × FANG, where FANG is angle of internal friction of matrix material. Compared to the intact rock elements, stiffness of fault elements is reduced, and that alters the stress state in and around the fault elements. As the reservoir simulation model does not consider the reactivated fractures or faults explicitly, it does not simulate the fluid diffusion process in the reactivated faults, which represents a limitation of the model. However, the reservoir modeling allows us to quantify the amount of additional fluid pressure transmitted during HF through the system of complex HFs and NFs from treatment wells to the interpreted fault zone. Since the conductivity of HFs and reactivated NFs is high, the pressure loss from the wellbore to the fault is therefore insignificant. By increasing the fluid pressure in the fault, the corresponding plastic deformation of the fault simulated, and the amount of pressure in the fault required for the fault reactivation is quantified. During 3D reservoir geomechanical modeling, the fluid pressure in the fault zone is increased by 5 MPa, 10 MPa, 15 MPa and 20 MPa in four time-steps, which helped to quantify the additional pressure required to cause plastic deformation in fault elements. Based on simulation result, we analyze orientation and magnitude of plastic normal and shear strain and plastic shear displacement in fault elements.

All the geological grids used by the different simulators are the same structured grids except the unstructured grid used in Intersect. The unstructured grid is generated based on the VISAGE grid and the fracture geometry simulated in the KINETIX grid, and it is used for flow simulation. The pre-injection stress simulation considers the fault; however, natural fractures are ignored. The post-injection stress simulation takes into account pressure changes, but natural fractures and the simulated hydraulic fracture network from previous steps are ignored. The fluid flow simulation considers the enhanced permeability from the simulated hydraulic fracture network, but not the natural fractures, which are not part of the hydraulic fracture network. The data transfer between simulators is carried out smoothly. The KINETIX grid is extracted from 3D geomechanical grid along with all estimated geomechanical and reservoir properties, stresses and pore pressure. Once HFM is completed, the production grid is generated that incorporates the information on HFs simulated in KINETIX. The pore pressure changes estimated in INTERSECT are incorporated in VISAGE™ 3D geomechanical model to estimate changes in stresses induced by pore pressure increase and evaluate the risk of fault reactivation. The time required to run a model depends on many factors, including size of a model, mesh size, number of elements, and hardware capacity. In our case, the modeling was conducted in a desktop Dell workstation, where runs in each different simulator have taken several hours up to 1–2 days, except for the reservoir modeling that had taken several days to a week.

## Results

### 3D static geomechanical model

The static Young’s modulus and Poisson’s ratio are the highest in the overlying and underlying carbonate rocks of the Wabamun Group and Swan Hills Formation (60–62 GPa and 0.29–0.33), and they are the lowest in the organic-rich shale of Duvernay Formation (25.4 GPa, 0.181). Unconfined compressive strength (UCS) values are approximately 100 MPa in the Duvernay Formation. Friction angle values are approximately 40° in the Duvernay shales.

The estimated vertical stress gradient in the Duvernay Formation is approximately 25–25.5 kPa/m, and formation pressure gradient is between 16 and 17 kPa/m. Horizontal stress gradients are the highest in carbonate rocks above and below the Duvernay Formation, with the minimum horizontal stress gradient of 22.8–25.2 kPa/m and maximum horizontal stress gradient of 25.2–28.8 kPa/m. The minimum horizontal stress gradient is 19.5–20.5 kPa/m and maximum horizontal stress gradient is 21.5–22 kPa/m in the Duvernay Formation. The orientation of the maximum horizontal stress was determined previously to be N43° E in the Fox Creek area and N45.5° E in the WCSB (Reiter et al. 2014; Shen et al. 2019a, b).

### Discrete fracture modeling

The DFN-1 model (Konstantinovskaya et al. 2021) is characterized by two vertical fracture sets, oriented parallel (N43° E) and orthogonal (N133° E) to SHmax (Fig. 5a). Fracture length was simulated under the power law distribution and the length of explicit fractures was defined to be from 10 to 1000 m in the DFN-1 model. The selected ranges are in agreement with fracture lengths estimated based on the average injection rate and fluid leakoff into the formation (MacKeen et al. 2019). Fracture intensity distribution was determined as fracture area over volume (P_32_) and it was set to be 0.05 m^2^/m^3^ in this model.

The DFN-2 model similarly includes two vertical fracture sets oriented parallel and orthogonal to SHmax (N43° E), and a third fracture set is oriented N23° E, which is 20° to SHmax (Fig. 5b). In all three fracture sets, the length of explicit fractures was defined to be from 10 to 100 m and fracture intensity P_32_ was set to be 0.01 m^2^/m^3^, using the same distribution laws as in the DFN-1 model.

### Hydraulic fracturing

**Fig. 6:**
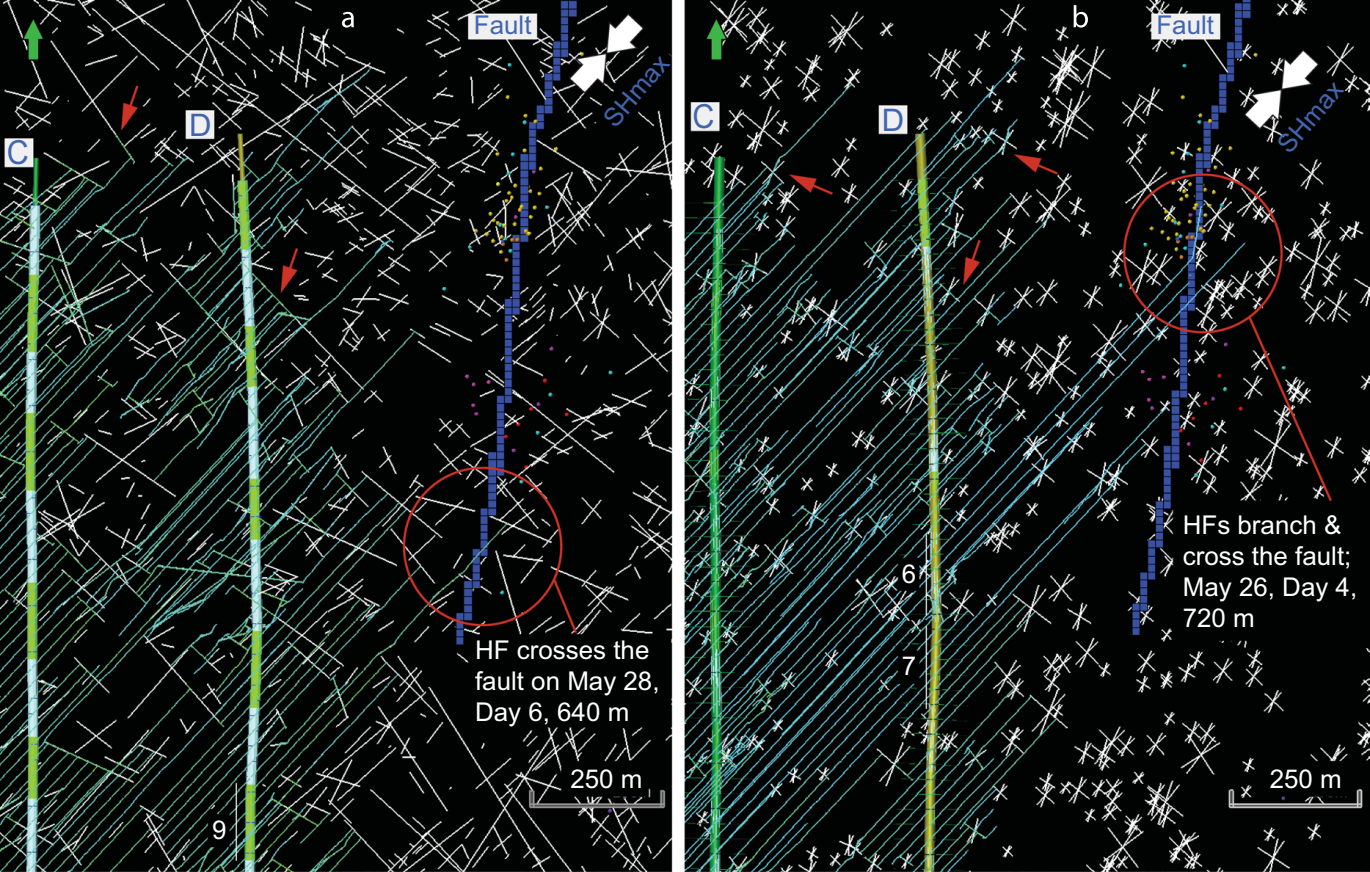
Geometry of simulated complex HFs of Case 1 (**a**) with two sets of NFs in DFN-1 model and Case 2 (**b**) with three sets of NFs in DFN-2 model. The HFs from stage 9 (**a**) or stage 6 (**b**) cross or branch the fault on the 6th or 4th day from the beginning of treatment of wells C and D, respectively. Red arrows indicate branching of HFs into orthogonal and oblique NFs. The displayed depth slice is parallel to a horizon located at the bottom of the Duvernay Formation. Fault elements (blue color) correspond to the fault location in 3D geomechanical grid. See color code in Fig. 2b for origin time of the induced earthquakes

*Case 1* HF simulation was carried out in horizontal wells C and D with natural fractures represented by DFN-1 model (Fig. 6a). Simulated HFs interact with NFs resulting in complex hydraulic fracture networks. The dominant HFs are mostly oriented NE-SW, parallel to SHmax (N43° E). Commonly, these HFs are arrested at the intersection with orthogonal NFs oriented NW–SE, parallel to Shmin (N133° E). In some cases, fluid continues to propagate along dilated or sheared orthogonal NFs until next available natural fracture with preferred NE–SW orientation parallel to SHmax, along which HFs continue to grow laterally away from the well. Less frequently, HFs cross orthogonal NFs. Half-length of simulated HFs parallel to SHmax in Case 1 ranges from 30 to 350 m with a mean value of 250–350 m (Fig. 6a) that correlates well with the previous estimation of median value of 309 m, standard deviation15.9 m in the study area (McKean et al. 2019). One HF of stage 9 in well D exceptionally propagates to the NE over a distance of 640 m; it reaches and crosses the fault on May 28, the 6th day from the beginning of fracturing of wells C and D (Fig. 6a).

*Case 2* HFM was conducted in horizontal wells A, B, C, and D and it uses DFN-2 as the natural fracture model (Fig. 6b). Similar to Case 1, HFs generally propagate in the NE-SW direction, parallel to SHmax. The NE-SW HFs are again arrested by orthogonal NFs oriented NW–SE. In some cases, HFs branch and run along dilated or sheared orthogonal fractures oriented parallel to Shmin (N133° E), but most frequently HFs turn and align with sheared oblique NFs that are oriented at 20° to SHmax (N23° E). In fewer cases, HFs cross orthogonal NFs. Half-length of HFs oriented parallel to SHmax in Case 2 varies from 100 to 800 m, and mostly from 200 to 500 m. Shorter natural fractures and lower fracture intensity P_32_ in DFN-2 favor lateral propagation of HFs in Case 2, if compared to Case 1.

In Case 2, one of the three HFs of stage 6 crossed the fault zone, the other two turned into the fault zone in the fault strike direction toward the northern segment of the fault and the microseismic events. These three HFs reached the fault over the distance of 720 m on May 26, the 4th day from the beginning of fracturing of wells C and D (Fig. 6b). The obtained result for the DFN-2 model correlates well with the 4-days’ time interval elapsed between the beginning of hydraulic fracturing in wells C and D and induced earthquakes associated with reactivation events (Fig. 2b). Three HFs of Stage 6 (May 26) could result in fluid pressure increase in the fault zone and subsequent shear slip along its northern segment, where reactivation microseismic events were recorded on May 26–29 (Figs. 2b, 6b). In addition, two hydraulic fractures from Stage 7 (May 27) stopped propagating very close to the fault location (Fig. 6b) and could contribute to pressure front transfer to the southern segment of the fault zone, where cluster of microseismic events occurred from May 31 to June 2 (Fig. 2b).

Both in Case 1 and Case 2 of HFM, majority of simulated HFs are vertically limited between the tops of the Lower Ireton and Swan Hill Formations with an average height ranging from ~ 35 m to ~ 200 m. The productive (propped) fracture height ranges between 20 and 100 m and most of the propped fractures are located within the Duvernay Formation. The generally good containment of simulated HFs within the Duvernay Formation can be explained by the stress contrast predicted by 3D finite element model (FEM) at the top and bottom of the formation (Konstantinovskaya et al. 2021). These results match median fracture height of 94.6 m, standard deviation 5.9 m previously estimated in the area (McKean et al. 2019) that ranges between 0.1 and 0.5 times of fracture length.

**Fig. 7:**
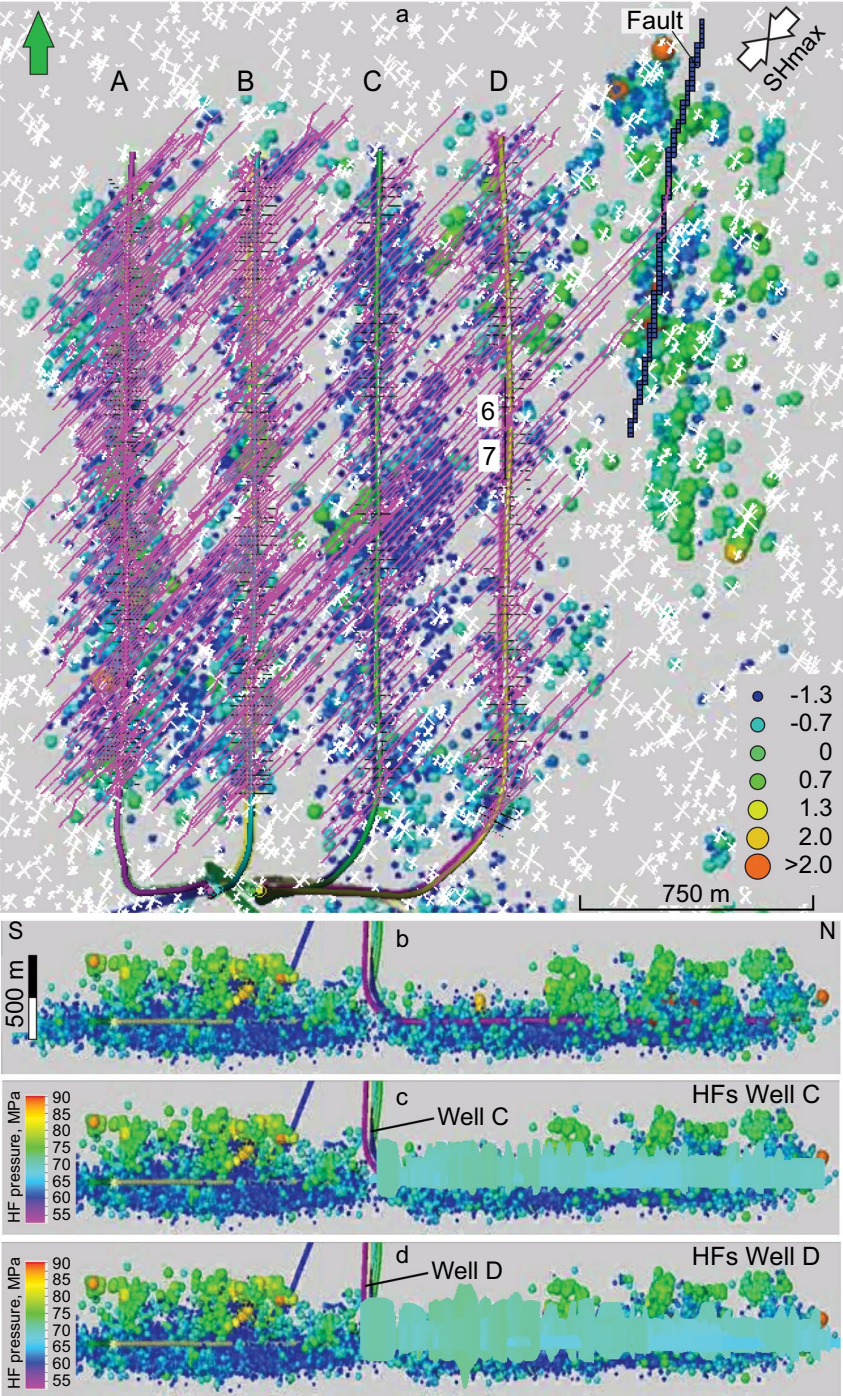
Map (**a**) and cross-sections (**b**–**d**) showing distribution of microseismic events occurred during treatment of lateral wells in Pad X. HFs (pink lines in a, green color in **c**, **d**) are simulated for Case 2 with three sets of NFs in DFN-2 model (white lines). HFs, NFs and fault elements (dark blue cells) are displayed in a slice parallel to a horizon located at the bottom of the Duvernay Formation. Microseismic events are shown by variable size and color corresponding to magnitude (blue small, orange high). See text for more explanation

The results of HFM are compared to the spatial distribution of microseismic event clouds (Fig. 7) and calibrated against measured bottomhole treatment pressure. The microseismic events of low magnitude in the vicinity of the wells that are shown on the map of microseismicity by blue color (Fig. 7a) are associated with hydraulic fracturing stages and line up with SHmax orientation and the simulated HFs (Fig. 7a). The spatial extents of small microseismic events around the wells and simulated HFs are similar in lateral and vertical extent (Fig. 7a, c, d). The larger-magnitude events that are shown on the map by green to orange colors and aligned as the N-S linear zone to the east of the treatment wells in the study area were interpreted as mostly induced reactivation events associated with strike-slip displacement along a fault (Schultz et al. 2017; McKean et al. 2019). The focal mechanism of one of the larger-magnitude events, the earthquake of 3.9 Mw that occurred on June 13, 2015 in the vicinity of the interpreted fault, corresponds to a dextral strike slip (Figs. 1a, 12) (R. Wang et al. 2016; Schultz et al. 2017). Unfortunately, there are no focal mechanism solutions for the larger-magnitude events in the vicinity of the wells and south of the fault (Fig. 7a). Given that the moment magnitude is proportional to the area of the discontinuity that slipped and the amount of slip (Zoback and Gorelick 2012), these larger-magnitude microseismic events could also be interpreted as slips of larger fractures compared to micro-fractures. The good containment of simulated HFs within the Duvernay Formation is supported by the microseismic event clouds (Fig. 7c, d). On the N–S cross-section, standard microseismic related events are concentrated within the reservoir zone of the Duvernay Formation, where the horizontal wells were landed, while most of the reactivation events are located above it (Fig. 7b). The depths of reactivation events were calibrated against perforation shots identified in the processing.

The uncertainty of the event location ranges from 0.1 m for large‐magnitude events to 900 m for small‐magnitude events near the study area boundary (McKean et al. 2019). However, the spatial uncertainties for the horizontal plane (x and y directions/dimensions for uncertainty ellipsoids) for the hydraulic fracturing induced events are small relatively to the dimensions of the hydraulic fracture, averaging at Average-X-Uncertainty = 3.76 m and Average-Y-Uncertainty = 3.00 m (Appendix 2), based on the data taken for the selected stage available in on-line supporting materials from (McKean et al. 2019). Uncertainty for vertical direction is higher, which is usual for microseismic monitoring technique, and averaging at Average-Z-Uncertainty = 21.85 m (Appendix 2). For the purpose of our study however, vertical direction of microseismic positioning is less critical since the vertical fracture propagation in the hydraulic fracturing simulations is mostly constrained by the vertical geomechanical profile in the area of interest.

Propped width of simulated HFs in Cases 1 and 2 ranges between 1 and 5 mm. Fracture width at wellbore is about 3–7 mm, and average fracture propped width is about 1.4–2.8 mm.

The most extended HFs in well D, which propagated to the NE and reached the fault, are characterized by highest propped fracture surface area and propped fracture length, and lowest propped fracture height and lowest propped fracture width, if compared to HFs from other stages. For the HF of stage 9 in Case 1, these parameters are 21,260 m^2^, 816 m, 25 m, and 2.83 mm, respectively. For three HFs of stage 6 in Case 2, these parameters are 23,398–28,179 m^2^, 539–630 m, 43–44 m and 1.02–1.41 mm, respectively.

**Fig. 8:**
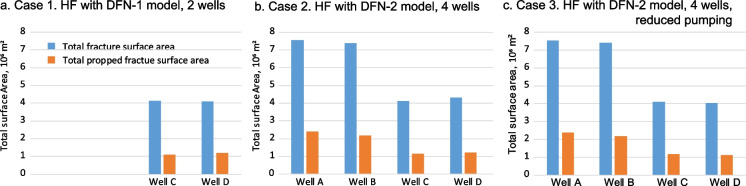
Total fracture surface area and propped fracture surface area (10^6^ m^2^) simulated in HFM for wells C and D (Case 1), and wells A, B, C, and D (Cases 2 and 3). See Table 1 for corresponding values

Total and propped fracture surface area (FA) in wells C and D of Cases 1 and 2 are close (Table 1, Fig. 8). Total FA in well C is slightly higher in Case 1 than in Case 2, because HF of wells A and B likely created stress shadow reducing FA in this well in Case 2. Total FA in well D in Case 1 is slightly less than in Case 2 because HFs in Case 2 propagate farther in presence of shorter and fewer natural fractures of DFN-2 (Fig. 6).

**Table 1:** Total and propped fracture surface area (FA) in HFM simulation for Cases 1–3

Case 1: HF with DFN-1, 2 wells C and D					
	well A	well B	well C	well D	Total 2 wells C-D
Total FA, m^2^			4,132,024	4,101,813	8,233,837
Propped FA, m^2^			1,089,202	1,201,548	2,290,750

Case 2: HF with DFN-2, 4 wells A, B, C, and D						
	well A	well B	well C	well D	Total 2 wells C-D	Total 4 wells
Total FA, m^2^	7,536,214	7,392,578	4,111,877	4,320,471	8,432,348	23,361,140
Propped FA, m^2^	2,391,942	2,182,527	1,138,399	1,196,007	2,334,406	6,908,875

Case 3: HF with DFN-2, 4 wells A, B, C, and D; reduced pumping in stages 6–7 of well D						
	well A	well B	well C	well D	Total 2 wells C-D	Total 4 wells
Total FA, m^2^	7,536,214	7,392,578	4,094,615	4,035,323	8,129,938	23,058,730
Propped FA, m^2^	2,391,942	2,182,527	1,181,318	1,106,713	2,288,031	6,862,500

**Fig. 9:**
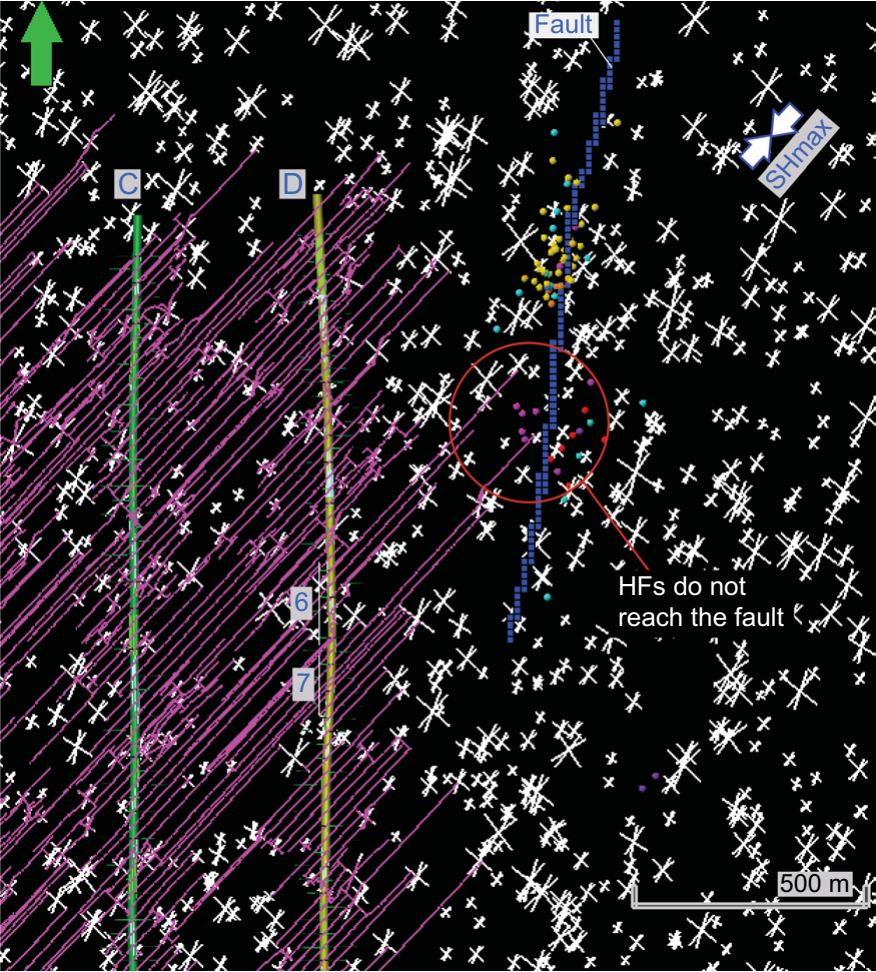
Geometry of simulated complex HFs in Case 3 of optimized pumping schedule in stages 6 and 7 of well D. HFs interact with three sets of NFs in DFN-2 model. The HFs from stage 6 do not reach the fault. The displayed depth slice is parallel to a horizon located at the bottom of the Duvernay Formation. Fault elements (blue color) correspond to the fault location in 3D geomechanical grid. See color code in Fig. 2b for origin time of the induced earthquakes

*Case 3* Sensitivity study, HF simulations are carried out for wells A, B, C and D using DFN-2 model, similar to Case 2, but pumping schedule in well D is optimized to prevent hydraulic fractures reaching the fault and therefore to minimize the risk of induced seismicity. The pumping schedule in well D is modified for stages 6 and 7 to decrease fluid volume and proppant mass that would result in reduction of the eastward HFs propagation from these stages and to prevent HFs reaching the fault located to the east of the well. The fluid volume is reduced from 2690 to 491 m^3^ for stage 6, and to 2252 m^3^ for stage 7. The proppant mass was reduced from 301 to 10 T for stage 6 and to 200 T for stage 7. As a result, simulated HFs from stages 6 and 7 do not reach the fault (Fig. 9). This represents one possible scenario of simulated HFs not reaching the fault. When multiple realizations of DFN models are used in the HF simulations, the results may not be the same. Therefore, in designing a HF job near existing faults, multiple HF simulation scenarios with multiple DFN realizations are required to minimize the probability of HFs reaching the faults.

Total fracture surface area and total propped fracture surface area in well D decrease in Case 3 if compared to Case 2 from 4.32 × 10^6^ m^2^ to 4.04 × 10^6^ m^2^ (6.6%) and from 1.20 × 10^6^ m^2^ to 1.11 × 10^6^ m^2^ (7.5%), respectively (Table 1, Fig. 8). In well C, total fracture surface area is slightly reduced but propped fracture surface area is increased, likely due to interaction of HFs between wells C and D. Fracture surface area in wells A and B remain the same in both cases. For all 4 horizontal wells, fracture surface area decreases by 1.3% and propped fracture surface area decreases by 0.67%.

The modeling results of the sensitivity case and estimations of the impact of injected fluid volume and proppant mass on propped fracture surface area per stage show that this approach helps to optimize pumping schedule while preserving overall pad performance and at the same time minimize the risk of fault shear slip reactivation and induced seismicity.

### Reservoir simulations

**Fig. 10:**
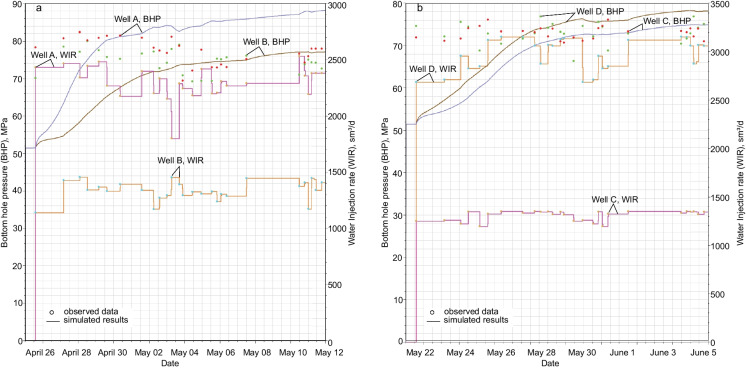
Observed data (dots) of bottomhole pressure (BHP) and water injection rate (WIR) and corresponding simulated results (solid lines) in wells A and B (**a**) and C and D (**b**) during HF

According to the available pumping data, wells A and B were fractured in zipper mode from April 25 to May 11, 2015 with an average pumping rate of 13.2–13.5 m^3^ per minute (Fig. 10a). In Well A, 27 stages were fractured with an average fluid volume of 1509 m^3^ per stage. There were 27 stages of hydraulic fractures in Well B and the average fluid volume per stage is 86 m^3^. In wells C and D, 15 stages were fractured also in zipper mode from May 22 to June 5, 2015 with an average pumping rate of 14.3–14.6 m^3^ per minute (Fig. 10b). The average fluid volume per stage in Wells C and D are 1373 m^3^ and 2824 m^3^, respectively.

The injection of the fracturing fluid to the horizontal wells and transport of the fluid in the fracture networks was simulated using unstructured simulation grid. During the reservoir simulations, we used different settings involving observed data of bottomhole pressure (BHP) and water injection rate (WIR) for historical control and/or BHP constraint (Appendix 1). In the final case presented here, we applied observed water injection rate as historical control mode, while no constraint on bottomhole pressure was imposed. The simulated bottomhole pressure in horizontal wells A–D is characterized by a reasonably good correlation with the observed BHP data (Fig. 10). However, BHP in Well A is a little affected by lack of continuous observed data in the mid- to later stages of injection and therefore had not enough observed data points to constrain the simulated result. We still consider the BHP match in well A good as it meets the average BHP of the well.

**Fig. 11:**
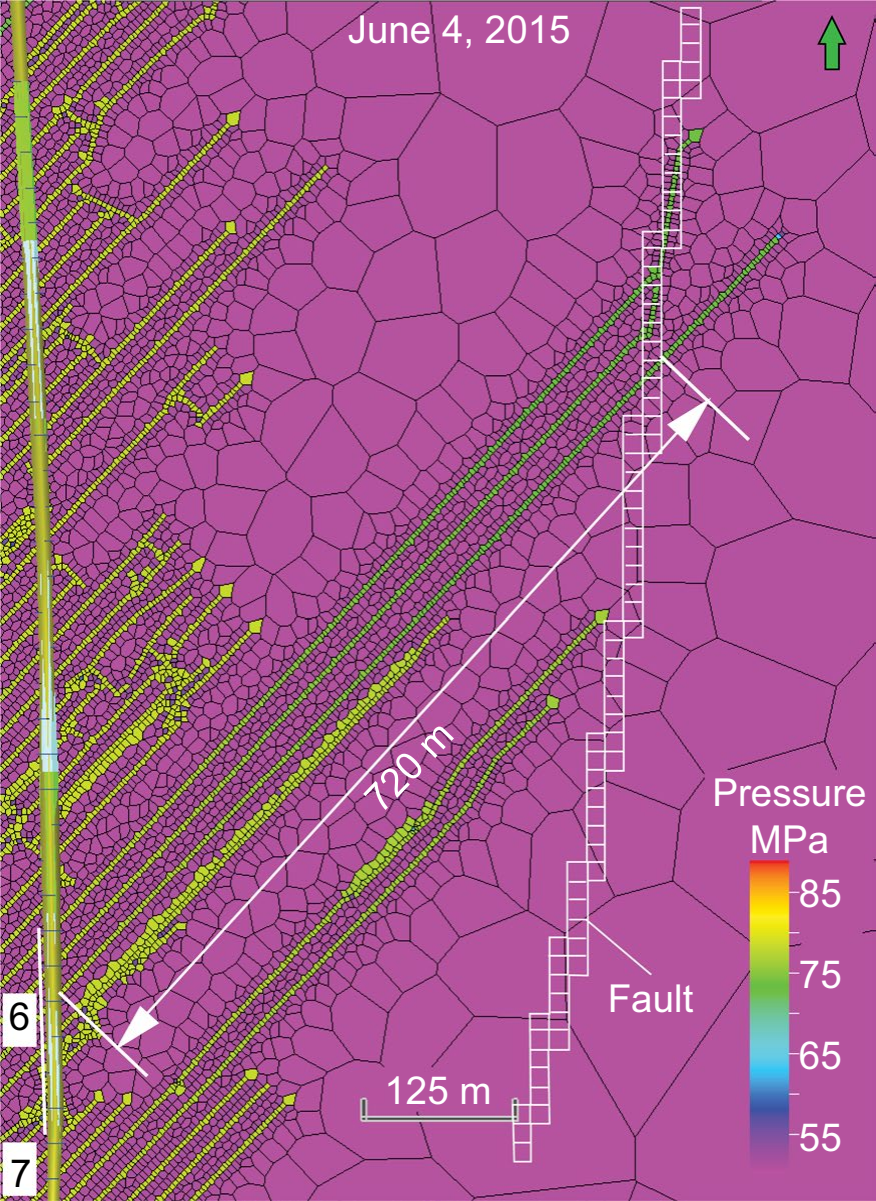
Simulated pore pressure in complex HFs–NFs in well D on June 4, 2015, at the end of injection phase. Note the increase of fluid pressure by 20 MPa is transmitted to the fault location through three HFs of stage 6 that cross and branch the fault on May 26, 2015, the 4th day from the beginning of treatment of wells C and D

The assumed high conductivity of HFs and reactivated NFs explains the insignificant pore pressure loss from the wellbore to the fault. According to reservoir simulations for the case HF with DFN-2 model, additional pressure of 20 MPa is transmitted during HF through the complex HFs–NFs from well D to the interpreted fault zone over the distance of 720 m (Fig. 11) on May 26, within 4 days after the beginning of treatment of wells C and D on May 22. Besides hydraulic fractures, open natural fractures that are connected to the hydraulic fractures and the fault zone could also have contributed to the pressure increase in the fault zone.

Further steps of history match of reservoir simulation model for a production stage and actual gas production for the studied wells for the first several months of production, if made available, along with microseismic data will be helpful to provide insights into hydraulic fracture connectivity and effective fracture lengths in the study area.

### 3D coupled reservoir geomechanical modeling

To understand the impact of fluid pressure increase in the fault zone on fault deformation, 3D one-way coupled reservoir geomechanical simulations were performed (Konstantinovskaya et al. 2021). An increase of fluid pressure in a fault zone results in a decrease of the normal effective stress acting on a fault plane that results in higher tendency for the fault plane to slip (Morris et al. 1996).

**Fig. 12:**
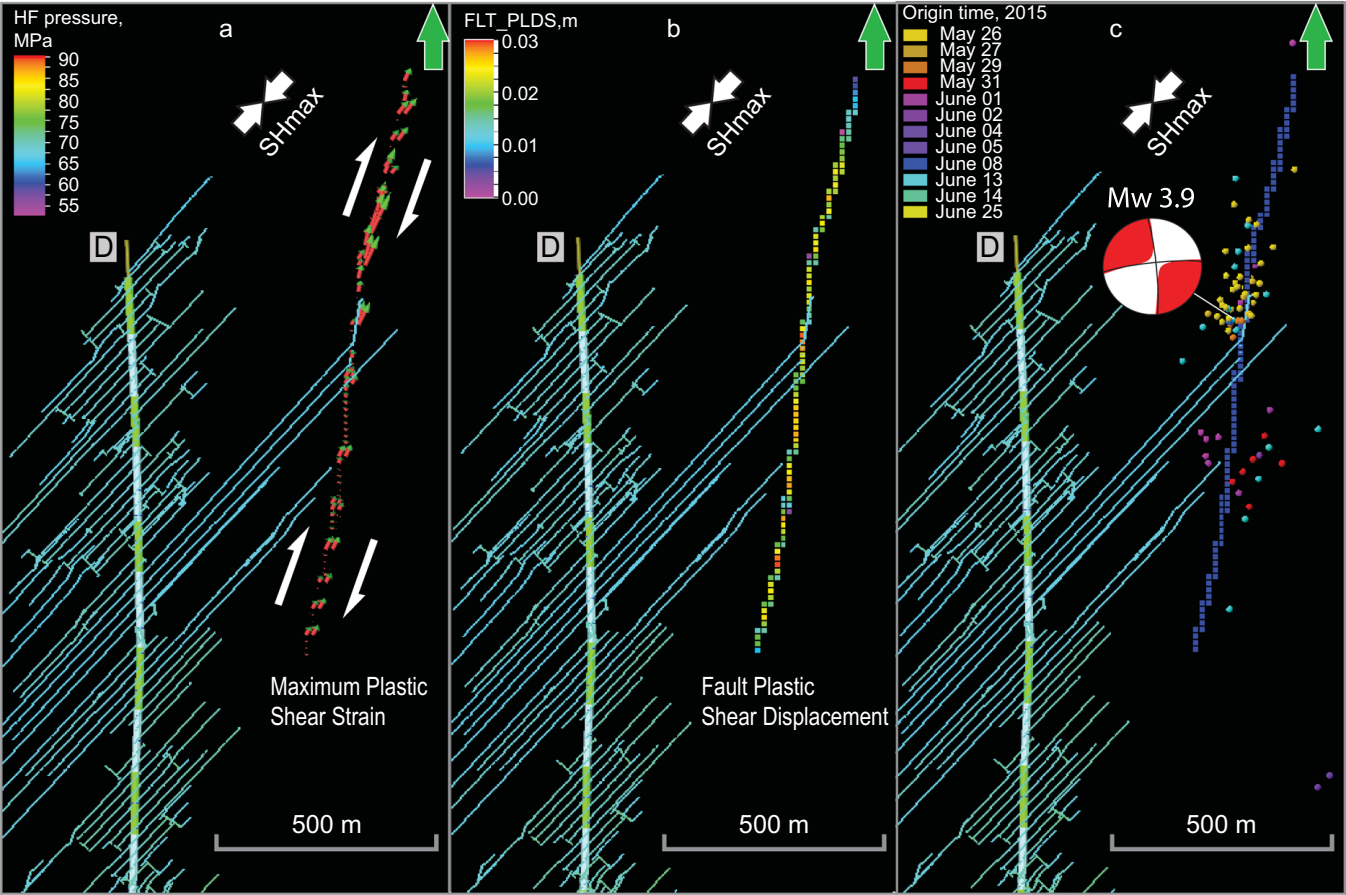
**a**, **b** Orientation of maximum plastic shear strain (**a**) and magnitude of fault plastic shear displacement (**b**) simulated in fault elements by FEM in 3D reservoir geomechanical modeling on May 26, 2015, at time-step of fluid pressure increase in the fault zone by 20 MPa; (**c**) induced earthquakes in the fault zone area. Size of vectors in (**a**) is proportional to the magnitude of the plastic shear strain. HFs and fault elements are displayed in a slice parallel to horizon located at the bottom of the Duvernay Formation. The focal mechanism for the earthquake occurred on June 13, 2015 is shown after (Wang et al. 2016; Schultz et al. 2017)

In 3D geomechanical model, the plastic shear strain initially occurs in fault elements at the additional fluid pressure of 5 MPa in the fault zone at the depth of the Duvernay Formation, where the initial reservoir pressure was the highest (Konstantinovskaya et al. 2021). The area of shear slip progresses upward and downward in fault elements as the fluid pressure in the fault zone increases by 10, 15 and 20 MPa (Fig. 12) and it occurs in the depth interval of the Duvernay and Ireton Formations at the last time-step. The orientation of vectors of plastic shear strain in fault elements is consistent with dextral strike slip mode along the fault (Fig. 12a). Plastic shear deformation is higher along the northern and southern segments of the fault, which are optimally oriented at 33° to SHmax (Fig. 12a), where most of the induced earthquakes were recorded (Fig. 12c). The relay zone is oriented at 43° to SHmax and it is characterized by lower magnitude of plastic shear strain (Fig. 12a) and very few to no microseismic events (Fig. 12c).

The maximum plastic shear displacement in fault elements is 3.08 cm (Fig. 12b). The upper bound of the moment release Mo of 7.73 × 10^14^ Nm was roughly estimated (Konstantinovskaya et al. 2021) as the product of fault maximum plastic shear displacement (0.0308 m) × fault slip area (1.4 km × 0.6 km) × shear modulus of fault material (30 GPa). A more accurate estimation of Mo of 6.47 × 10^14^ Nm was obtained by using a summation of the moment release for finite elements along the fault. These two simulation-based estimates are close to the independent value observed from radiated seismic energy (Hanks and Kanamori 1979), which is 7.9 × 10^14^ Nm for the Mw 3.9 event (Zoback and Gorelick 2012).

Uncertainty remains whether the plastic deformation simulated in the fault elements in this study may result in sudden slip along the fault surface, which is usually the source of reactivation microseismic events. However, given that induced earthquakes were recorded on the day, when increased fluid pressure front reached the fault, it can be suggested that high shear strain and shear displacement as simulated in fault elements could have induced sudden slips along the fault.

## Discussion

Our previous attempt of HFM simulation without introducing HF–NF interaction has shown that planar bi-wing HFs propagate for ∼1500 m in the NE direction and ∼800 m in the SW direction (Konstantinovskaya et al. 2021). That is far over the fracturing area and microseismic events clouds and does not respect interwell spacing of 200–320 m established for the optimal reservoir development in the area. Additionally, high natural fracture density was reported in the Duvernay Formation (Kleiner and Aniekwe 2019) that partially explains prolific productivity of the organic-rich mudstones with very low matrix permeability. The background tectonic fracturing in the Duvernay Formation is considered significantly greater than in most other low permeability reservoirs in North America, and its nature and age would be an interesting subject for further investigation.

The interaction of simulated HFs with NFs represented by DFN may vary in 2D and 3D HF modeling depending on many parameters, including scale of modeled fractured rock masses, in-situ stress field and reservoir pressure, mechanical response, mesh-dependent framework, hydromechanical simulation code (Smart et al. 2014; Weng 2015; Rutqvist et al. 2018; Sun and Schechter 2018; Tutuncu et al. 2018).

The HFM results presented in this study (Fig. 6, 9) are similar to complex hydraulic fracture networks simulated in the Duvernay Formation and reported earlier (Ramanathan et al. 2015; Ferrer et al. 2020). Based on the results of HF simulations with DFN-1 (Case 1) and DFN-2 (Cases 2 and 3) of this study (Fig. 6), the primary factors that control lateral growth of HFs are the in-situ stress field, maximum length limit of explicit fractures and fracture intensity (P_32_) in DFN models. NFs in the DFN-2 model with lower fracture intensity (0.01 m^2^/m^3^) and shorter length (max 100 m) create fewer barriers that arrest HFs, if compared to NFs in the DFN-1 model (0.05 m^2^/m^3^, 1000 m). Additional stress shadow from the HFs of wells A and B could also have contributed in driving HFs from wells C and D toward the fault.

The modeled HFs, that propagate NE–SW parallel to the SHmax orientation, are most frequently either arrested by orthogonal NFs or branch orthogonal or oblique NFs, similar to other simulation results (Weng et al. 2011; Gu et al. 2012; Wu et al. 2012; Alimahomed et al. 2020; Zhang et al. 2020). It is considered that when fluid pressure in fracture is higher than fracture closure pressure, NF opens up in tension. When fluid pressure is below the fracture closure pressure, fracture can fail in shear (Weng 2015). When HF is arrested by NF, NF may fail in shear and slip in case when the NF interface is weaker than the rock matrix; otherwise, it can be opened in tension. It is likely that under the settings of present-day strike-slip stress regime and increasing fluid pressure during HF simulated in our study, the NW–SE orthogonal NFs, being parallel to Shmin, open in tension, while oblique NFs oriented at 20° to SHmax may slip and open in shear dilation. Less frequently, the HFs simulated in our study directly cross the orthogonal NW–SE NFs (Fig. 6). The crossing of a NF by a HF likely happens when a NF has strong mechanical bonding and/or subject to high normal stress (Weng 2015). In this study, the NFs oriented NW–SE are subject to high normal stress because they are orthogonal to SHmax, or maximum in situ stress, under the strike-slip stress regime. Our results are in line with the consideration that the combination of opening in tension and shear dilation control enhanced permeability of NFs and “stimulated volume” around the treatment wells (Li and Dusseault 2016). In-situ stress field, orientation of NFs relative to in situ stresses, rock mass mechanical properties and fracture interface bonding are the factors that contribute to the dominant mechanism of dilation of NFs in complex HFs–NFs networks during HF.

The HFM simulation results indicate that complex HFs–NFs networks facilitate hydraulic communication to the fault over a distance range from 640 m (Case 1) to 720 m (Case 2), or even farther (816 m half-length of the HF that crosses the fault in Case 2), depending on the DFN input. The simulated insignificant pore pressure loss from the wellbore to the fault is explained by high conductivity of HFs and NFs. Unfortunately, there is no direct observed data from a monitor well that would help to quantify pressure or aperture change in HFs–NFs in the study area. The 1/r pressure decay behavior is usually expected causing rapid pressure drop away from an injector, as it was simulated in poroelastic homogeneous reservoir with permeability 0.3 mD for the case of a single-well injection with constant rate 900 m^3^/d and pressure drop of about 2 MPa at the distance of 1000 m from the injector (Segall and Lu 2015). However, the unconventional reservoir of organic-rich shales of the Duvernay Formation considered in this study is very tight with very low matrix permeability ranging from 10 to 150 nD (Kleiner and Aniekwe 2019; Konstantinovskaya et al. 2021). The HFs and NFs represent the main contribution to flow in unconventional reservoirs (Cramer 2008; Li and Dusseault 2016). It has been shown that a widespread zone of fracturing can enhance drainage of the Barnett Shale reservoir by creating permeability channels (Gale et al. 2007). The log–log plot of producing rate vs time often shows a long-term linear trend that supports that flow is dominated by an anisotropic fracture-enhanced zone in the vicinity of a primary fracture trend (Cramer 2008). Additionally, the fracture-enhanced flow in unconventional reservoir is supported by the case of lateral well communication that has been observed in situ in the Horn River Basin within distance of above 1 km using natural tracers (Fu and Dehghanpour 2020). It is also in the agreement with cases of induced seismicity observed a few days later after the beginning of hydraulic fracturing at a distance up to 1–1.5 km from the HF stimulation in the Kaybob and Red Deer areas (Wang et al. 2020; Schultz and Wang 2020; Schultz et al. 2020). The pore pressure transfer was simulated along 1 km-long fracture corridors oriented N30° E parallel to the fast S-wave orientation and induced earthquakes clusters (Igonin et al. 2021). In general, our modeling results support that fluid pressure increase caused by HF, especially in overpressured reservoirs, and transmitted along complex fracture networks to pre-existing faults may trigger either earthquake slip or aseismic slip along the faults (Eaton and Schultz 2018; Eyre et al. 2019, 2022; Schultz et al. 2020). Indeed, the finite-discrete element numerical simulations and laboratory triaxial experiments have shown that fracturing at meso-scale can result in enhancing localized permeability in tight shales with 0 to 5 orders of magnitude (Hyman et al. 2016). More complex, dynamic permeability evolution was observed in laboratory experiments of fracture permeability with periods of permeability enhancement induced by hydro-shearing interchanging with periods of slow continuous permeability loss caused by chemo-mechanical compaction during periods of pre- and post-stimulation hold phases (Yildirim et al. 2020).

## Conclusions

The research study involving the workflow of DFN modeling, HFM and reservoir simulations, and 3D coupled reservoir-geomechanical modeling was conducted in unconventional reservoir of organic-rich mudstones of the Upper Devonian Duvernay Formation in the area located south of Fox Creek area, Alberta. The obtained results helped us to investigate the interaction between NFs represented by DFN and HFs, evaluate the role of complex HFs–NFs networks in possible fluid flow path from the injection wells to the fault zone and estimate potential of fault shear slip reactivation to explain the occurrence of induced earthquakes (up Mw 3.9) during hydraulic fracturing of horizontal wells in May–June 2015.

Several mechanisms of HFs–NFs interaction were simulated. HFs are most frequently either arrested by NFs or branch into orthogonal or oblique NFs. Less frequently, HFs directly cross the NW–SE NFs. The geometry of NFs, in particular, maximum length of explicit fractures and fracture intensity, and in-situ stress regime likely provides major control on lateral growth of HFs. Stress shadow from earlier stimulated wells could also cause asymmetric propagation of later hydraulic fractures preferentially toward lower stress regions. The permeability of NFs involved in complex HFs–NFs networks is likely enhanced by the combination of opening in tension and shear dilation caused by increased fluid pressure in the overpressured reservoir during HF. In-situ stress field, orientation of NFs relative to in situ stresses, rock mass mechanical properties and fracture interface bonding are the factors that contribute to the dominant mechanisms of dilation of NFs in complex HFs–NFs networks during HF.

The reservoir simulations demonstrate that predicted lateral fluid migration can occur from the injection wells to the interpreted fault through the system of complex HFs–NFs over the distance of 720 m in 4 days, resulting in fluid pressure increase in the fault zone. The 3D coupled geomechanical simulation revealed that dextral shear slip started to occur along the fault at the depth interval of the Duvernay Formation, when fluid pressure in the fault zone increased by 5 MPa. At higher fluid pressure, the area of fault shear slip increased. The simulated plastic shear strain in fault elements correlates with the recorded microseismic events. This confirms that the recorded earthquakes in the study area were most probably associated with the dextral shear slip along the fault. The shear slip of the fault was caused by fluid pressure increase during hydraulic fracturing that was transmitted from the treatment wells to the fault through complex HFs–NFs networks in the Duvernay reservoir.

The modeling results of the HFM sensitivity case allowed us to demonstrate that reducing injected fluid volume and proppant mass in selected stages in one of the horizontal wells may help to control lateral HF propagation and prevent fault pressurizing. For the real hydraulic fracturing treatments, the same exercise of decreasing volume in selected stages could be done using the real-time microseismic fracturing monitoring and volumes adjustment in real time during the treatment in the vicinity of faults. As we did show in our study, minor volume adjustment on the pad scale can be used to minimize the risk of fault shear slip reactivation and induced seismicity while preserving overall pad performance.

## Figures and Tables

**Fig. 13:**
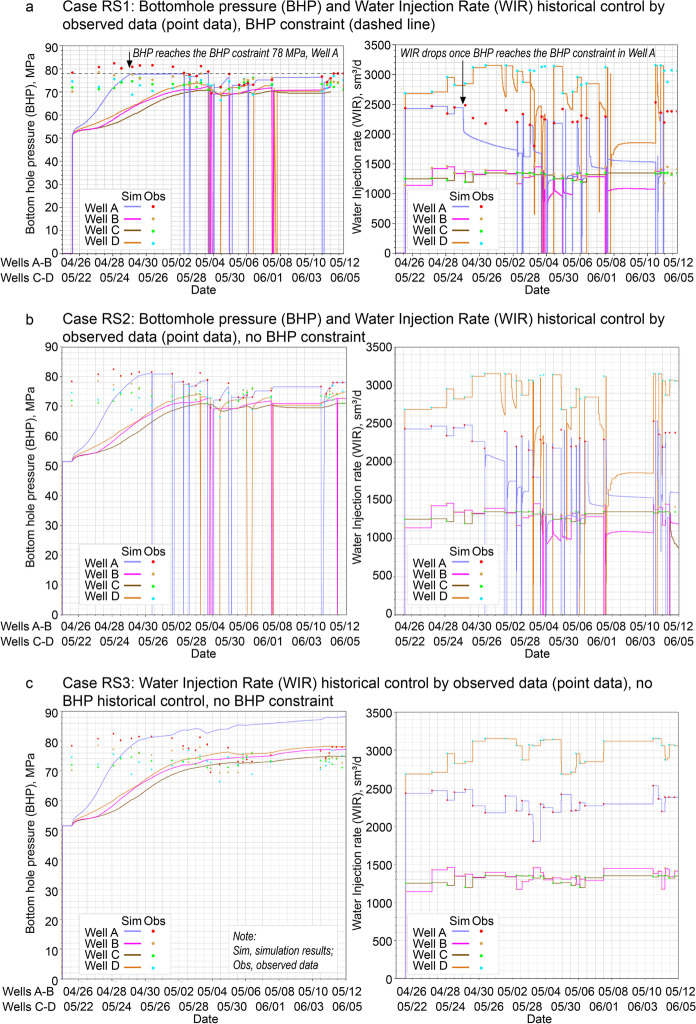
Three cases of reservoir simulations RS1-RS3 conducted under different settings. The bottomhole pressure (BHP) and water injection rate (WIR) observed in wells A–D during HF are shown by dots, the simulation results are shown by solid lines

**Fig. 14:**
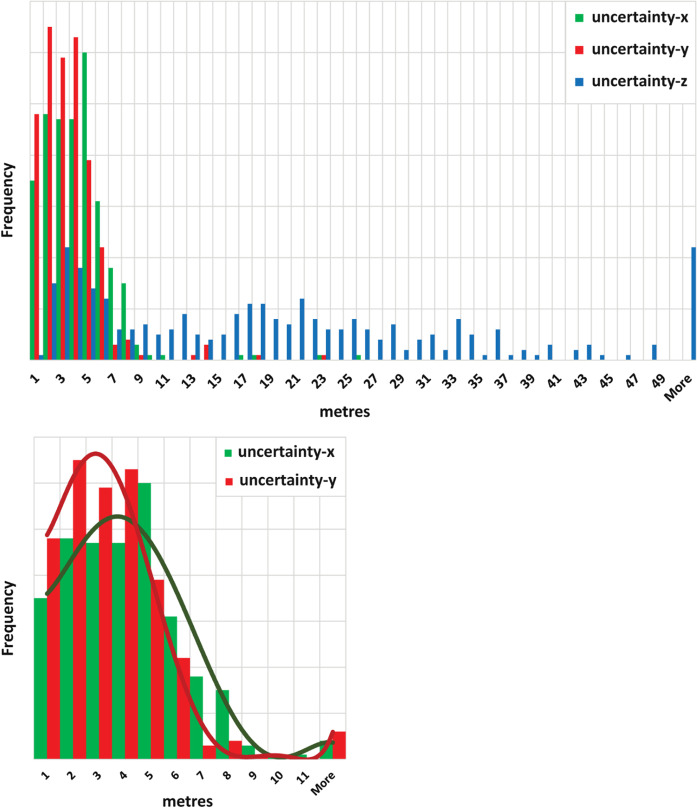
Frequency distribution histograms for x, y and z uncertainties in location of small-magnitude microseismic events for the selected stage

## Appendix 1

Several numerical reservoir models were built to account for observed data of the bottomhole pressure (BHP) and water injection rate (WIR). In Case RS1, the observed data of BHP and WIR for wells A–D and BHP constraint were used in the simulation (Fig. 13a). The BHP constraint was set equal to 78 MPa for well A, 80 MPa for well B, 77 MPa for well C, and 74 MPa for well D. In Case RS2, only observed data of BHP and WIR for wells A–D were applied, while no constraint on BHP was included in the settings of the simulation run (Fig. 13b). In Case RS3, only WIR observed data for wells A–D were applied in the simulation, neither BHP observed data, nor BHP constraint were used in this run (Fig. 13c).

The results of the simulation run Case RS1 demonstrate that once BHP in well A reaches the imposed BHP constraint of 78 MPa, the pressure stops to build up in the well, and WIR drops at the same time (Fig. 13a). Similarly, the WIR drops in wells A–D in Cases RS1 and RS2, when BHP tries to fit the observed BHP data (Fig. 13a, b). In Case RS3, presented in the paper (Fig. 10), the buildup of BHP in wells A–D is simulated with no historical control or constraint, only WIR observation data are used (Fig. 13c). The simulated BHP in horizontal wells A–D is characterized by a reasonably good correlation with the observed BHP data. However, the BHP buildup is higher in well A, if compared to other wells (Figs. 13c). It can be explained by the relationship of fracture geometry, total injected fluid volume and water injection rate (WIR). In well D, the engaged rock volume during hydraulic fracturing is greater than in well A because the length of some of HFs is greater, including those of stages 6 and 7 reaching the fault (Fig. 7). The lower stimulated rock volume (SRV) in well A results in higher BHP buildup in this well, if compared to well D, even though the total injected fluid volume and WIR in these wells are comparable.

## Appendix 2

The frequency distribution histograms for x, y and z uncertainties in location of small-magnitude microseismic events for the selected stage. The histograms are estimated based on the data available in the on-line supporting materials from (McKean et al. 2019) (Fig. 14).

## References

[CR1] Alimahomed F, Wigger E, Drouillard M, Rosas GG, Kolbeck C (2020) Impact of pore pressure on modeled hydraulic fracture geometry and well spacing in the East Duvernay Shale Basin, Canada. In: SPE/AAPG/SEG unconventional resources technology conference 2020, URTeC 2020, URTEC-2019–516, pp 1–39. 10.15530/urtec-2019-516

[CR2] Atkinson G, Eaton DW, Ghofrani H, Walker D, Cheadle B, Schultz R, Shcherbakov R (2016). Hydraulic fracturing and seismicity in the Western Canada Sedimentary Basin. Seismol Res Lett.

[CR3] Becerra Delmoral R, Konstantinovskaya E (2020) Multimineral petrophysical analysis to solve for complex lithologies of unconventional reservoirs: a case study from The Duvernay Shale in Alberta, Canada. GSA 2020. 10.1130/abs/2020AM-354933

[CR4] Cramer DD (2008). Stimulating unconventional reservoirs: lessons learned, successful practices, areas for improvement. Soc Pet Eng Unconv Reserv Conf.

[CR5] Dunn L, Gordon K, Houle M (2013) Fifty shades of grey: utilizing "conventional" sedimentology and sequence stratigraphy to unlock rock quality to reservoir quality relationships in the liquids rich Duvernay Shale play. GeoConvention Integr 1–3

[CR6] Eaton DW, Schultz R (2018). Increased likelihood of induced seismicity in highly overpressured shale formations. Geophys J Int.

[CR7] Eaton DW, Igonin N, Poulin A, Weir R, Zhang H, Pellegrino S, Rodriguez G (2018). Induced seismicity characterization during hydraulic-fracture monitoring with a shallow-wellbore geophone array and broadband sensors. Seismol Res Lett.

[CR8] Eyre TS, Eaton DW, Garagash DI, Zecevic M, Venieri M, Weir R, Lawton DC (2019). The role of aseismic slip in hydraulic fracturing–induced seismicity. Sci Adv.

[CR9] Eyre TS, Samsonov S, Feng W, Kao H, Eaton DW (2022). InSAR data reveal that the largest hydraulic fracturing-induced earthquake in Canada, to date, is a slow-slip event. Sci Rep.

[CR10] Ferrer GG, Faskhoodi M, Zhmodik A, Li Q, Mukisa H (2020) Completion optimization of child wells in a depleted environment, a Duvernay example. In: Society of Petroleum - SPE Canada Unconventional Resources Conference 2020, URCC 2020; SPE 199986. 10.2118/199986-ms

[CR11] Fothergill P, Boskovic D, Schoellkopf N, Murphy P, Mukati MA (2014) Regional modelling of the Late Devonian Duvernay Formation, western Alberta, Canada. In: Society of petroleum - SPE/AAPG/SEG unconventional resources technology conference. 10.15530/urtec-2014-1923935

[CR12] Fu Y, Dehghanpour H (2020). How far can hydraulic fractures go? A comparative analysis of water flowback, tracer, and microseismic data from the Horn River Basin. Mar Pet Geol.

[CR13] Gale JFW, Reed RM, Holder J (2007). Natural fractures in the Barnett Shale and their importance for hydraulic fracture treatments. Am Assoc Pet Geol Bull.

[CR14] Gu H, Weng X (2010) Criterion for fractures crossing frictional interfaces at non-orthogonal angles. In; 44th US rock mechanics symposium and 5th US-Canada rock mechanics symposium, Salt Lake City, Utah, USA, p. ARMA 10–198

[CR15] Gu H, Weng X, Lund J, MacK M, Ganguly U, Suarez-Rivera R (2012). Hydraulic fracture crossing natural fracture at nonorthogonal angles: a criterion and its validation. SPE Prod Oper.

[CR16] Haege M, Zhmodik A, Boskovic D, Ramanathan V, Hoffart T, Li Q (2015). Duvernay fracturing: from microseismic monitoring to unconventional fracture model construction. GeoConvention.

[CR17] Hamilton WN, Price MC, Langenberg CW (1999) Geological map of Alberta, Map 236, scale 1: 1,000,000, Edmonton, Alberta. Retrieved from https://ags.aer.ca/publication/map-236

[CR18] Hyman JD, Jiménez-Martínez J, Viswanathan HS, Carey JW, Porter ML, Rougier E, Karra S (2016). Understanding hydraulic fracturing: a multi-scale problem. Philos Trans R Soc A Math Phys Eng Sci.

[CR19] Igonin N, Verdon JP, Kendall J, Eaton DW (2021). Large-scale fracture systems are permeable pathways for fault activation during hydraulic fracturing. J Geophys Res Solid Earth.

[CR20] Kleiner S, Aniekwe O (2019) The Duvernay Shale completion journey. In: Society of petroleum - SPE Kuwait oil & gas show and conference 2019, KOGS 2019, vol SPE198070, pp 13–16. 10.2118/198070-ms

[CR21] Konstantinovskaya E, Li Q, Zhmodik A, Ibelegbu C, Schultz R, Shipman T (2021). Lateral fluid propagation and strike slip fault reactivation related to hydraulic fracturing and induced seismicity in the Duvernay Formation, Fox Creek area, Alberta. Geophys J Int.

[CR22] Leshchyshyn T, Thomson J, Larsen C (2016) Duvernay proppant intensity production case study and frac fluid selection. In: SPE annual technical conference and exhibition, vol SPE181687, pp 1–25. 10.2118/181687-ms

[CR23] Li R, Dusseault MB (2016) Shale gas geomechanics and insights into hydraulic fracturing stimulation. Fifth EAGE Shale Work, Catania, Italy. 10.3997/2214-4609.201600416

[CR24] Li Q, Michi O, Boskovic D, Zhmodik A, Faskhoodi M, Ferrer G, Ramanathan V et al (2020) Geomechanical characterization and modeling in the Montney for hydraulic fracturing optimization. In: Society of petroleum - SPE Canada unconventional resources conference, URCC 2020; SPE 199978. 10.2118/199978-ms

[CR25] Lyster S, Corlett HJ, Berhane H (2017) Hydrocarbon resource potential of the Duvernay Formation in Alberta – update. In: Alberta energy regulator/Alberta geological survey open file reports 2017–02. Retrieved from http://ags.aer.ca/OFR_2017_02.html

[CR26] McKean SH, Priest JA, Dettmer J, Eaton DW (2019). Quantifying fracture networks inferred from microseismic point clouds by a Gaussian mixture model with physical constraints. Geophys Res Lett.

[CR27] Morris A, Ferrill DA, Henderson DB (1996). Slip-tendency analysis and fault reactivation. Geology.

[CR28] Preston A, Garner G, Beavis K (2016) Duvernay reserves and resources report: a comprehensive analysis of Alberta’s foremost liquids-rich shale resource, Edmonton, Alberta. Retrieved from https://static.aer.ca/prd/documents/reports/DuvernayReserves_2016.pdf

[CR29] Ramanathan V, Boskovic D, Zhmodik A, Li Q, Ansarizadeh M, Perez Michi O, Garcia G (2015) A simulation approach to modelling and understanding fracture geometry with respect to well spacing in multi well pads in the Duvernay - A case study. In: Society of petroleum - SPE/CSUR unconventional resources conference, vol SPE175928. 10.2118/175928-ms

[CR30] Reiter K, Heidbach O (2014). 3-D geomechanical-numerical model of the contemporary crustal stress state in the Alberta Basin (Canada). Solid Earth.

[CR31] Reiter K, Heidbach O, Schmitt D, Haug K, Ziegler M, Moeck I (2014). A revised crustal stress orientation database for Canada. Tectonophysics.

[CR32] Rutqvist J, Figueiredo B, Hu M, Tsang CF (2018). Continuum modeling of hydraulic fracturing in complex fractured rock masses. Hydraul Fract Model.

[CR33] Schultz R, Wang R, Gu YJ, Haug K, Atkinson G (2017). A seismological overview of the induced earthquakes in the Duvernay play near fox creek, Alberta. J Geophys Res Solid Earth.

[CR34] Schultz R, Skoumal RJ, Brudzinski MR, Eaton DW, Baptie B, Ellsworth W (2020). Hydraulic fracturing-induced seismicity. Rev Geophys.

[CR35] Schultz R, Wang R (2020). Newly emerging cases of hydraulic fracturing induced seismicity in the Duvernay East Shale Basin. Tectonophysics.

[CR36] Segall P, Lu S (2015). Injection-induced seismicity: Poroelastic and earthquake nucleation effects. J Geophys Res Solid Earth.

[CR37] Shen LW, Schmitt DR, Haug K (2019). Quantitative constraints to the complete state of stress from the combined borehole and focal mechanism inversions: Fox Creek, Alberta. Tectonophysics.

[CR38] Shen LW, Schmitt DR, Schultz R (2019). Frictional stabilities on induced earthquake fault planes at Fox Creek, Alberta: a pore fluid pressure dilemma. Geophys Res Lett.

[CR39] Smart KJ, Ofoegbu GI, Morris AP, McGinnis RN, Ferrill DA (2014). Geomechanical modeling of hydraulic fracturing: Why mechanical stratigraphy, stress state, and pre-existing structure matter. Am Assoc Pet Geol Bull.

[CR40] Sun J, Schechter D (2018). Pressure-transient characteristics of fractured horizontal wells in unconventional shale reservoirs with construction of data-constrained discrete-fracture network. SPE Prod Oper.

[CR41] Thiercelin MJ, Plumb RA (1994). Core-based prediction of lithologic stress contrasts in East Texas formations. SPE Form Eval.

[CR42] Tutuncu AN, Bui B, Suppachoknirun T (2018). An Integrated study for hydraulic fracture and natural fracture interactions and refracturing in shale reservoirs. Hydraul Fract Model.

[CR43] Wang R, Gu YJ, Schultz R, Kim A, Atkinson G (2016). Source analysis of a potential hydraulic-fracturing-induced earthquake near Fox Creek. Alberta Geophys Res Lett.

[CR44] Wang J, Li T, Gu YJ, Schultz R, Yusifbayov J, Zhang M (2020). Sequential fault reactivation and secondary triggering in the march 2019 red deer induced earthquake swarm. Geophys Res Lett.

[CR45] Weng X, Kresse O, Cohen C, Wu R, Gu H (2011) Modeling of hydraulic fracture network propagation in a naturally fractured formation. In: SPE hydraulic fracturing technology conference, vol SPE 140253

[CR46] Weng X (2015). Modeling of complex hydraulic fractures in naturally fractured formation. J Unconv Oil Gas Resour.

[CR47] Wu R, Kresse O, Weng X, Cohen C, Gu H (2012) Modeling of interaction of hydraulic fractures in complex fracture networks. In: Society of petroleum *- *SPE hydraulic fracturing technology conference 2012, vol SPE152052. 10.2118/152052-ms

[CR48] Yildirim EC, Im K, Elsworth D (2020). The influence of fault reactivation on injection-induced dynamic triggering of permeability evolution. Geophys J Int.

[CR49] Zhang Q, Zhang X-P, Sun W (2020). A review of laboratory studies and theoretical analysis for the interaction mode between induced hydraulic fractures and pre-existing fractures. J Nat Gas Sci Eng.

[CR50] Zoback MD, Gorelick SM (2012). Earthquake triggering and large-scale geologic storage of carbon dioxide. Proc Natl Acad Sci U S A.

